# Evolving paradigms for natural-product drug discovery

**DOI:** 10.1093/nsr/nwac075

**Published:** 2022-04-22

**Authors:** Geoffrey A Cordell

**Affiliations:** Natural Products Inc., USA; College of Pharmacy, University of Florida, USA

## Abstract

Natural products are an essential aspect of global human health. Integration of contemporary technologies based on the Fourth Industrial Revolution (4IR) and the Quintuple Helix is needed to discover additional metabolites to be developed sustainably for the broad and unmet range of human healthcare needs.

Natural products and their derivatives have remained a reliable source of new pharmaceutical agents in the past 30 years [1]. For the future, however, new paradigms are requisite for the more high-profile, sustainable and focused exploration of nature, employing the contemporary technologies to address global disease needs [2]. The first of these is to shift from the Western ‘big pharma’ paradigm to one in which the initiatives are responsive to global patient-centered needs. To respond to the clinical urgency, approval systems are needed for a new class of standardized, safe and effective natural medicinal agents. Approval implies accessibility and there is a prevalent myth that the registered medicines (pharmaceuticals or traditional medicines) will always be there. That can only occur after the notion of ‘medicines security’ (availability, affordability and a safe and effective product consistency) is adopted by the World Health Organization [2].

The core premise of the Quintuple Helix initiative towards the Sustainable Development Goals is local and international collaboration between governments, academia and industry to focus and enhance research and product development, followed by integrated knowledge accumulation, culminating in overarching social ecology and focused on population needs [3]. This is a major shift from the investigator-initiated research paradigm. Any broad-based and collaborative drug-discovery program based on natural resources represents compromises within a strategy developed acknowledging the funding support for information systems, acquisition, taxonomy, chemistry and biology.

Collaboration is *the* fundamental aspect of discovery programs and requires high quality, intensity, reliability and consistency from all the participants and their programs for success. Such efforts are underpinned by negotiated agreements for intellectual property rights (IPR) sharing locally and at the sample source, providing compensation strategies for access to resources and publication authorship arrangements for the group.

Sustainability in drug discovery is not a choice. It is the contemporary wisdom that will allow future generations to thrive. Promoting sustainable philosophies and practices, including resource acquisition and experimentation, through ecopharmacognosy [4] is therefore vital. Long-term planning for sustainable sourcing is essential as the impacts of encroaching climate change and biodiversity loss are experienced. Medicinal plant gene banks and resource assessments are necessary to maintain critical medicinal plant supplies.

Integration of the seven contemporary technologies relating to natural products based on the Fourth Industrial Revolution (4IR) embrace the utilization of large data sets for artificial intelligence (AI) and machine learning (ML), robotics, additive manufacturing, biotech applications in discovery and development, nanotechnology for drug delivery and remote detection systems [5]. Immutable research data acquisition and analysis are essential to protect IPR and patent claims. Establishing a balance between ML, *in silico* metabolite prognostications, robotic processing of extracts, network pharmacology and the human interpretation of AI-generated outcomes is a critical element of contemporary integrated discovery programs.

The consolidation and global accessibility of databanks of traditional knowledge and botanical, chemical and biological information on natural products through a coordinated international effort are necessary [6]. New discovery initiatives must be based on well-defined niches of environmental and human and animal health needs, and avoid duplicative studies.

Enhanced reproducibility in natural-product research requires new paradigm thinking: as a taxonomist, know precisely what organism is being acquired and processed; as a chemist, know what has been characterized; and as a biologist know, ‘at the time of the test’, what chemical (or matrix) is being evaluated.

ML-driven and PCR-driven taxonomic identification is becoming an essential component for sustainable, reproducible sourcing and interfaces inexorably with the application of metabolomics and molecular networking for discerning biosynthetic pathways. Geolocations matter, since an ‘organism’ is not an organism; the name does not define the metabolome. Indeed, metabolic flux changes based on many exogenous parameters.

Bioactive metabolites that possess broad biological activities or are widely distributed in plants must be identified during the early discovery stages [7,8]. Terrestrial and marine organisms are characteristically associated with a plethora of parasitic or supportive microorganisms, which evince a separate, possibly potent, metabolite profile. Dereplication through an informatics–chemo–bioassay-linked system can identify known bioactives in each of these instances to potentiate isolation resources. ‘Old’ compounds can be important leads for new applications, having the potential advantage of an established safety profile, depending on the delivery system.

Awakening and defining ‘silent’ biosynthetic gene clusters in fungal and bacterial genomes are unexplored discovery sanctuaries for disclosing new metabolites [9]. Structure diversity can be modulated through gene manipulation of biosynthetic clusters and new enzymes characterized for chemical synthesis, possibly avoiding expensive, non-recyclable reagents. *In silico* binding studies can define new targets for the synthetic modification of bioactive natural products.

Developing robust, sensitive, rapid and cheap bioassays that preferentially target relevant new mechanisms is essential to identifying new bioactive scaffolds. Basic discovery programs should commence taxonomically, chemically and biologically at the point of acquisition in the field or marine environment. Microfluidic biosensors for in-field extract evaluation and hand-held devices offer rapid detection technology applications. Searching for synergistic and adjuvant compounds or metabolites that overcome drug resistance is a valuable discovery initiative. Establishing the breadth and depth of the genomic impact of a bioactive metabolite through network pharmacology is essential to distinguish true ‘leads’ from ‘hits’ with respect to effectiveness and potential toxicity.

Continuing education is critical. Broad-based, advanced degree programs are an integral aspect of training young scientists to conduct interdisciplinary and multidisciplinary, highly collaborative, focused research programs for bioactive natural products for future development. Training programs for established scientists are necessary as philosophies, techniques and instrumentation, including robotics, evolve. Team leaders should understand when to halt a research initiative or change direction based on critical program-assessment criteria.

Natural-product drug-discovery programs must contribute globally to addressing the chasm of unmet disease needs and evolve strategies based on sustainable natural resources. Identifying the diseases in which patient needs prevail should occur through dedicated collaborative programs whose success is based on scientific excellence and potentiates the strengths and activities of government, academia and industry. Integration of 4IR technologies is essential for effective natural-product drug discovery. Intellectual territories and egos must be set aside to promote collaborative research to ameliorate significant human healthcare needs for the quality, safety, efficacy, consistency and accessibility of all nature-derived medicinal agents [10].
